# The Aquarium Sign: Another Opportunity for Detection of Perforated Viscus

**DOI:** 10.5811/cpcem.2019.1.41582

**Published:** 2019-03-05

**Authors:** Matthew Gorgone, Timothy P. O’Connor, Michael Lu

**Affiliations:** University of Rochester Medical Center, Department of Emergency Medicine, Rochester, New York

## CASE PRESENTATION

A 61-year-old male with history of alcohol abuse and presumed cirrhosis presented to the emergency department with generalized weakness and right facial droop. He was found to be profoundly hypotensive and hypothermic with subsequent rapid decompensation requiring intubation and continuous norepinephrine infusion. Given the presence of ascites, we performed a diagnostic paracentesis that showed 9,787 nucleated cells per microliter with abundant intra- and extra-cellular bacteria. Intravenous vancomycin and piperacillin-tazobactam was started, but he was too hemodynamically unstable to travel to computed tomography to evaluate for perforation. A point-of-care ultrasound revealed air bubbles and debris actively bubbling (“aquarium sign”) through ascites and intraperitoneal A-lines indicative of pneumoperitoneum (Video). Portable abdominal radiograph was suspicious for free air, and eventually the patient was taken to the operating room where surgeons found a ruptured gastric ulcer and distal ileum and cecum ischemia without frank necrosis.

## DISCUSSION

Any patient admitted to the hospital with ascites secondary to cirrhosis should undergo diagnostic paracentesis to rule out spontaneous bacterial peritonitis (SBP).^1^ But distinguishing SBP from secondary causes such as viscus perforation can be difficult, as the classic symptoms of peritonitis do not often occur in patients with ascites because of the separation between visceral and parietal peritoneum.^2^ Differentiating these etiologies is critical due to high mortality of secondary bacterial peritonitis without surgical intervention, and high mortality of SBP if patients undergo unnecessary exploratory laparatomy.^2^,^3^ Many ultrasound techniques to detect pneumoperitoneum have been described, including assessment of the perihepatic space, movable gas, and peritoneal line enhancement, but assessment of these can be challenging to inexperienced sonographers.^4^

While movable gas is more easily visualized in patients with ascites, free fluid also provides a unique opportunity to visualize active air seepage from perforated bowel, as seen in our case. To our knowledge, this has not been previously described.

**CPC-EM Capsule**

What do we already know about this clinical entity?

*Multiple findings are associated with pneumoperitoneum that can be detected on ultrasound, but these findings can be subtle*.

What is the major impact of the image(s)?

*Visualizing air rising to the top of ascites on ultrasound is an easily seen finding and is indicative of perforation*.

How might this improve emergency medicine practice?

*Detection of free air on ultrasound as part of the assessment of critically ill cirrhotic patients can expedite surgical consultation*.

## Supplementary Information

Video Ascites is visualized using a curvilinear transducer with the probe marker oriented toward the patient’s head. The image is obscured by what appears to be an “A’” profile reverberation artifact, which is displaced superiorly as pressure is applied to the probe, suggesting movable air within the peritoneal cavity. Next, a phased array transducer is used with the probe marker oriented toward the patient’s head. Hyperechoic air bubbles are visualized rising to the top of the ascites.
